# Atomistic Simulation on the Twin Boundary Migration in Mg under Shear Deformation

**DOI:** 10.3390/ma12193129

**Published:** 2019-09-25

**Authors:** Shichao Song, Yu Wang, Yang Wang, Xi Wang

**Affiliations:** 1 CAS Key Laboratory of Mechanical Behavior and Design of Materials, Department of Modern Mechanics, University of Science and Technology of China, Hefei 230027, China; songsc@mail.ustc.edu.cn (S.S.);; 2 School of Mechanical, Electronic and Control Engineering, Beijing Jiaotong University, Beijing 100044, China; wangxi@bjtu.edu.cn

**Keywords:** Magnesium, {1012} twin, simple shear, twin boundary migration mechanism

## Abstract

In this paper, the {$10\bar{1}2$} twinning and detwinning was studied by molecular dynamics simulation under different shear directions and strain rates. The results showed that the twin was thickened under [$\bar{1}011$] shear direction and shrunken with shearing in the opposite direction. The critical resolved shear stress of {$10\bar{1}2$} twin boundary migration increased with the increase of the strain rate. By analyzing the atom’s displacement, it was concluded that the {$10\bar{1}2$} twin migration was achieved by both the shear and the atomic shuffling. Every atom would be affected by the shear, and different shear directions would cause opposite move directions, which led to twinning or detwinning. The atom shuffling was only used for adjusting the glide twin boundary and mirror-symmetric twin boundary structure evolution.

## 1. Introduction

Twin boundary (TB) is a special kind of two-dimensional planar defect in crystalline materials. It separates two crystalline regions structurally as the mirror images of each other. The highly symmetrical discontinuity in structure can be produced by deformation or annealing. Inevitably, the structure and the properties of TBs deeply affect the mechanical properties of materials. Therefore, the thermodynamics and kinetics of twin boundary are central to understanding the properties and evolution of many materials systems.

Magnesium (Mg) and its alloys, due to their low density and high specific strength, have potential applications in automotive and aerospace industries [1]. However, low strength and ductility induced by their hexagonal closed-pack (HCP) structure are the major obstacles preventing Mg alloys being widely used. As is well known, the basal slip in Mg is the only preferable slip system to activate at room temperature, which is insufficient to accommodate general homogeneous plastic deformation according to the Von Mises criterion. Twinning is the other important deformation mechanism for Mg at room temperature [2,3,4,5]. In addition, since it can adapt to the deformation along the c-axis of HCP crystalline, the introduction of twinning deformation can significantly enhance the strength and ductility of Mg [6,7,8,9,10,11]. Among all the possible twinning systems, the relatively lower shear critical resolved shear stress (CRSS) of the {$10\bar{1}2$} extension twinning mode and the high mobility of the extension twin boundary (ETB) make it the most frequently observed during various deformation of Mg alloys [2,3].

Considerable attention has been focused on the improvement of mechanical properties via extension twinning induced by predeformation [6,7,9]. He et al. [6] introduced {$10\bar{1}2$} tension twin in a thin magnesium sheet by in-plane precompression in order to tailor the texture. They found that the room temperature stretch-formability of the pretwinned Mg alloy sheet was remarkably improved by about 50%. They concluded that the activation of the tensile twinning in the pre-twinned region could effectively accommodate the through-thickness strain during the stretch forming, which is assumed to be the main reason for the stretch-formability improvement. Chen et al. [7] investigated the strengthening mechanism of AZ31 magnesium alloy by {$10\bar{1}2$} extension twin during multipass compression. With the microstructure evolution analysis, they found that the {$10\bar{1}2$} extension twin dominated during multipass compression and led to dramatic grain refinement, which made great contribution to the enhancement of the yield and peak stress. From the above, the extension twin plays a crucial role in the mechanical behaviors of Mg alloys.

Additionally, it has also been found that magnesium alloys always exhibit the {$10\bar{1}2$} extension twinning-detwinning in some processes such as fatigue [12,13,14,15,16,17], pseudoelasticity [18,19,20], and damping capacity [21,22], etc. The twinning and detwinning dominate the deformation and subsequent reverse-deformation, which exhibit more complex nucleation and propagation mechanisms than those associated with dislocations. Extensive experimental, analytical, and computational studies have been devoted to understanding the behaviors of twinning and detwinning. Sarker et al. [8] focused on the twinning–detwinning of extruding Mg alloys in relation to the strain level and strain-hardening characteristics during the compressive deformation. It could be found that the activation of extension twinning would attribute to the presence of strong crystallographic texture and lead to the characteristic with decreasing strain-hardening rate. At the high strain level, the detwinning caused by the strong twin-dislocation interactions would result in an increasing strain-hardening rate. Xu [23] analyzed the twinning and detwinning mechanism and their roles in reducing the tension-compression asymmetry. They pointed out that the extension twin generated in the multidirectional pre-strain greatly influenced the deformation behaviors during the further deformation.

Beyond that, many efforts have been dedicated to the motion of ETB to reveal the mechanism of twinning and detwinning [24,25,26,27,28,29]. Liu et al. [29] observed the boundary migration of extension twin in single-crystal Mg under tension and compression by using in situ transmission electron microscopy (TEM). They found that there was no shear strain during the twinning and detwinning process. An atomistic simulation on a bicrystal model contained with a {$10\bar{1}2$} ETB under the tension deformation is performed to support the experiment results. Wang et al. [24] studied the boundary migration of serrated coherent extension twin boundaries. They found that serrated coherent TBs migrated through twin dislocations (TD) by gliding along coherent twin boundaries and climbing along basal–prismatic planes serrations. Li et al. [27] proposed a shuffling-dominated mechanism on the migration of {$10\bar{1}2$} ETB. They thought that the TB migration was achieved by the conversion of the basal planes to prism planes, and the structural reconstruction was mediated by atomic shuffling without the action of twin dislocations. 

Despite many efforts on probing the twinning and detwinning behaviors in Mg alloys, the TB migration mechanism was still unclear. Therefore, in this paper, the shear-couple grain migration method which had been successfully used in Face-Centered Cubic and Body-Centered Cubic materials [30,31,32,33,34,35,36,37] was carried out by molecular dynamics simulation. The influence of the strain rates and loading directions on the growth and shrinkage mechanism of {$10\bar{1}2$} extension twin are studied. The deformation mechanisms are analyzed according to the microstructure evolution.

## 2. Materials and Methods

In the investigation presented here, simulations were performed using molecular dynamics techniques. The simulation represents a small area of extension twin migrating through a deformed matrix under the external shear deformation. Compared to the extension twin microstructures observed in some experiments, the simulation cells are somewhat simplified, while still capturing the essential features of growth/shrinkage of the extension twin and allowing for a thorough analysis of the migration process of TB.

**Figure 1:**
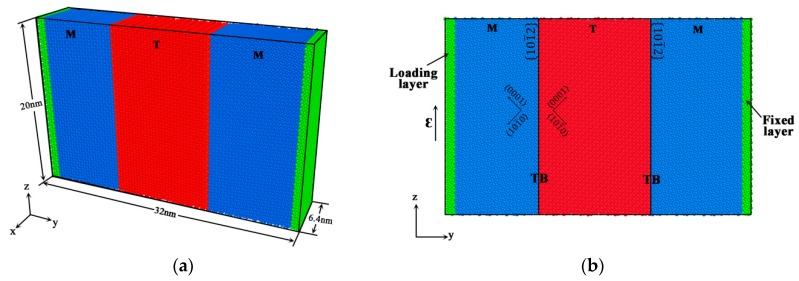
Illustration of the simulation model. {$10\bar{1}2$} Twin boundaries (TBs) are implied by black solid lines. Atoms in matrix are colored blue, and atoms in twin are colored red. The green atoms implied the loading and fixed layers. (**a**) 3D view; (**b**) Front view.

The potential used here is an embedded atom method developed by Sun [38], which has been successfully used to investigate many fundamental issues in Mg, such as the crack growth, transformation of twin boundary, etc. The initial setup consisted of three parts “patched” together in the simulation cell. Periodic boundary conditions were applied along the x- and z-directions; while the free boundary condition was applied along the y-direction. Figure 1 shows the simulation cell set up.

The simulation procedure included three stages: Setup of atoms, relaxation, and deformation, which was the same as other research work [31,32,33,37] on the boundary migration. During setup, atoms were arranged to produce crystallographic microstructure with two coherent ETBs existed between the matrix and twin blocks in cell. Initially, blocks of atoms in matrix arranged in perfect HCP crystal structures were with [$\bar{1}2\bar{1}0$] direction along the x-direction and [$\bar{1}011$] direction along the z-directions, respectively. The atoms of twin in the middle block were arranged in extension twin orientation with the matrix. The normal direction of the twin boundary between the matrix and twin blocks was along the y-direction. The simulation volume was chosen so that the crystal lattice blocks matched up perfectly at the periodic boundaries along the x- and z-directions. The size of the simulation cell was about 6.4 × 32 × 20 nm^3^. The number of atom layers in x-, y-, and z-direction were 40, 169, and 108, respectively; the total number of atoms was about 180,000. The twin block was in the middle of the simulation cell with about 11.7 nm thick in the y-direction.

The relaxation and deformation procedure were performed using a constant number of atoms, volume, and temperature (NVT) velocity Verlet MD algorithm by the LAMMPS [39] package. A Nose-Hoover thermostat algorithm was used for temperature control. The temperatures in the relaxation and deformation procedure were controlled at 0.1 K. The time step used in the calculation was 1 fs. The simulation cell was firstly relaxed for about 20,000 time steps without any applied stress and strain. Then, as shown in Figure 1b, the atoms with about 1 nm thick layer at the left end were applied displacement along the z-direction; while the atoms with 1 nm thick layer at the right end were fixed during the whole simulation process. The simple shear strain was γ_yz_ = d/L_y_, here, d and L_y_ were the loading-layer displacement and the length along y-axis, respectively. Three different strain rates were applied on the simulation cell: $\dot{\gamma }$ = 10^7^ s^−1^, $\dot{\gamma }$ = 5 × 10^7^ s^−1^ and $\dot{\gamma }$ = 10^8^ s^−1^. Meanwhile, another simulation along the opposite direction was conducted on the same cell to investigate the influence of loading direction on the twin boundary migration. The microstructural characteristic and its evolution rule were visualized by the OVITO 3.0.0 software [40]. Atoms were colored by their orientation calculated by the polyhedral template matching method [41].

## 3. Results and Discussion

**Figure 2:**
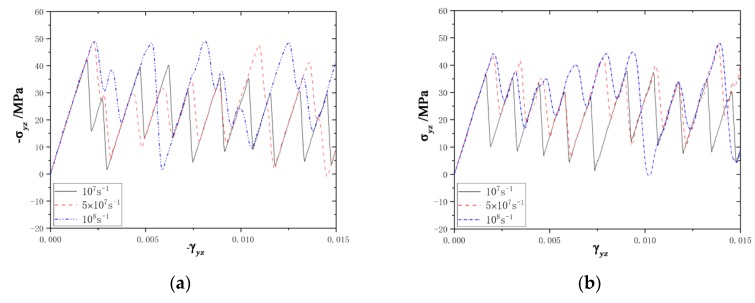
The stress-strain curves. (**a**) The curves under [$\bar{1}011$] shear direction. (**b**) The curves under [$10\bar{1}\bar{1}$] shear direction.

**Figure 3:**
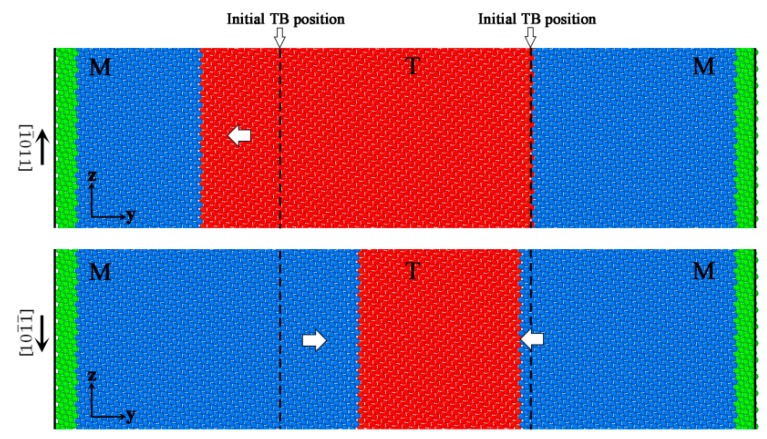
The atoms’ configurations under different shear directions with $\dot{\gamma }$ = 10^7^ s^−1^ when the amount of shear strain was 0.015. Atoms in matrix are colored blue, and atoms in twin are colored red. The green atoms implied the loading and fixed layers. The dotted lines are the initial TB positions.

The stress-strain curves are plotted in Figure 2. Stress tensors of the system were derived from atom stress, which was calculated by the compute stress/atom command [42] on LAMMPS. As shown in Figure 2, it showed similar stress-strain behavior for both loading conditions. The stress initially increased linearly during the elastic deformation for each curve, and then the stress fluctuated around an average value at all the strain rates. The atoms’ configurations with the different shear directions under the shear strain 0.015 and shear rate 10^7^ s^−1^ are plotted in Figure 3. It can be obviously seen that the twin grew up with shearing along the [$\bar{1}011$] direction and contracted with shearing along the opposite direction. With the twin boundary migrating, the elastic energy was dissipated, leading to the stress fluctuating around a constant value. Like the dislocation motion, TB migration was also an energy release process, corresponding to the process jumping from a Peierls valley to the next with stress drops [43]. Referring to the computing method of CRSS in dislocations, the CRSS was calculated by averaging the stress peaks during the fluctuation process [43,44,45]. When it was shearing along the [$\bar{1}011$] direction, the CRSS was about 34 MPa, 37 MPa, and 42 MPa, respectively, at strain rates of 10^7^, 5 × 10^7^, and 10^8^ s^−1^. For the opposite shear direction, the CRSS was 34 MPa, 37 MPa, and 41 MPa, respectively. The CRSS increased with the strain rates increasing, irrespective of the loading direction.

By measuring the migration distance, we could get the proportion relationship between the loading displacement and TB migration distance. The ratios are presented in Table 1. They were approximately equal under all shear rates. It could be concluded that the TB migration distance was only related to the loading displacement and had nothing to do with the strain rate. In HCP metals, the theoretical {$10\bar{1}2$} twinning shear is given by

(1) $$s=(3-v^{2})/\sqrt{3}v,$$

v = c/a, where a and c are the crystal constant of material. As for Mg, it could be that v = 1.628, indicating that the theoretical twinning shear was about 0.124. The ratio in Table 1 was close to the twin shear, which meant that the loading displacement might be applied to accommodate the shearing deformation during the TB migration process. In addition, the atom strain tensor was calculated by the atom strain method [46] in Ovito. The atomic deformation gradient tensor **F** was calculated for each particle based on the particle displacement vectors, and then atomic Green-Lagrangian strain tensor **E** was derived for each particle from

**E** = 1/2 (**F**^T^**F** − **I**) (2)

**Figure 4:**
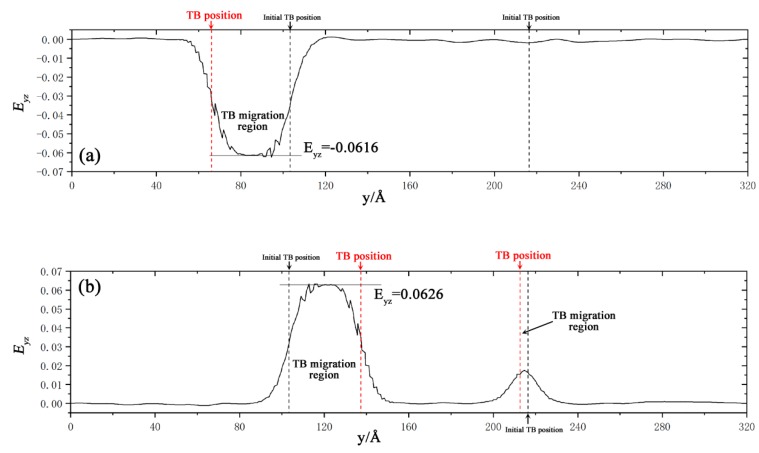
The E_yz_ distribution curves along y-axis under different shear directions with $\dot{\gamma }$ = 10^7^ s^−1^ when the amount of γ_yz_ was 0.015. (**a**) The curve shearing along [$\bar{1}011$] and (**b**) [$10\bar{1}\bar{1}$].

The E_yz_ distribution curves along the y-axis are plotted in Figure 4, when the amount of γ_yz_ was 0.015. The curves showed that the main strain was concentrated in TB migration region. The E_yz_ was −0.0616 in the main migration region with shear along [$\bar{1}011$] and it was 0.0626 with the opposite shear direction. The twin shear was calculated by s = 2E_yz_, they were about −0.123 and 0.125 in the dominating migrated region. By determining from the deflection of marker lines experimentally, Molodov [28] also got the amount of the twin shear of 0.126 in the twin region. The twin shears in simulation and experiment were both in excellent agreement with the theoretical value, which indicated that the shear was an indispensable part in the twin migration.

**Figure 5:**
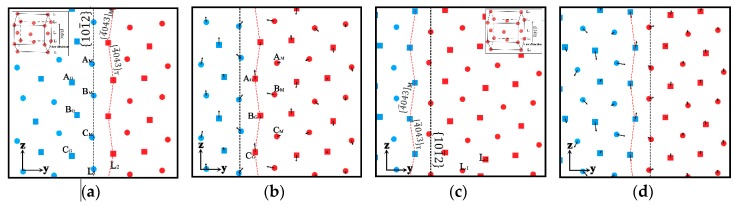
The atoms’ structure and actual displacement before and after TB migration. (**a**,**b**) The maps sheared along [$\bar{1}011$] direction. (**c**,**d**) The maps sheared along [$10\bar{1}\bar{1}$] direction. The atoms in twin and matrix are marked in red and blue color, respectively. The atoms located on different {$\bar{1}2\bar{1}0$} planes are represented by the solid circles and solid squares, which are labeled as L_1_ and L_2_, respectively. Atom displacement is represented by black arrows. TBs are expressed by black and red dotted lines.

The TB migration mechanism under different shear directions were studied. The microstructural evolution of atoms were analyzed under the shear rate $\dot{\gamma }$ = 10^7^ s^−1^ (Figure 5). The atoms in twin and matrix were marked in red and blue color, respectively. The atoms located on different {$\bar{1}2\bar{1}0$} planes were represented by the solid circles and solid squares, which were labeled as L_1_ and L_2_, respectively. The twinning boundary on different {$\bar{1}2\bar{1}0$} planes were expressed by the black and red dotted line, respectively. By observing the microstructural characteristics in different layers (Figure 5a), the atoms on the twin boundary of L_1_ layer were nearly along a straight line and in a mirror-symmetric twin relationship. Therefore, the twin boundary in this layer was recorded as a mirror-symmetric twin boundary (MSTB). Meanwhile, the atoms on the twinning boundary of L_2_ layer were in a zigzag line and in a glide twin relationship [47,48], which was recorded as glide twin boundary (GTB). As shown in Figure 5b,d, the displacement vector arrows of atoms during the twinning boundary migration process were plotted. It could be found that the displacement evolution of the atoms in the two types of TB was different during the migration process.

**Figure 6:**
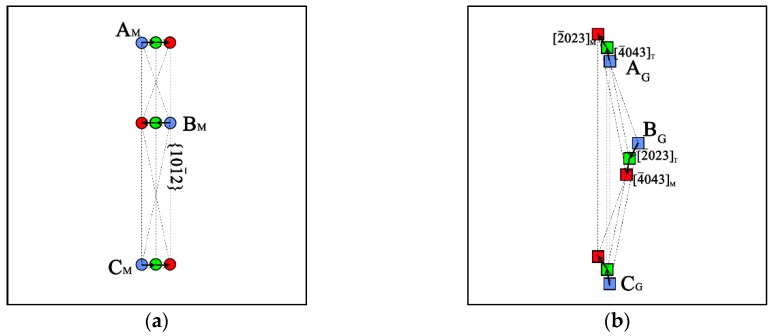
The TB structure evolution process on different {$\bar{1}2\bar{1}0$} planes. (**a**) The mirror-symmetric TB (MSTB) on L_1_ layer. (**b**) The glide TB (GTB) on L_2_ layer. The blue, red, and green symbols represent the atoms in matrix, twin, and TB structure, respectively. The arrows show the atom movement directions.

For a better illustration of the atoms’ motion during migration process, the movement of six atoms, labeled in Figure 5a, in the twin boundary of L_1_ layer and L_2_ layer was tracked. In Figure 6a, it is shown that in the L_1_ layer, the movements of the atoms A_M_, B_M_, and C_M_ were nearly perpendicular to the normal of {$\bar{1}2\bar{1}0$} plane, which was transmitted from the matrix state (blue circle) to the TB state (green state) and then the twin state (red state). However, the movements of the atoms A_G,_ B_G_, and C_G_ in L_2_ layer presented a different motion path (Figure 6b). The atoms A_G_ in the matrix state (blue square) firstly moved along the ${\left[\bar{4}043\right]}_{T}$ direction to the TB state (green square), and then moved along the ${\left[\bar{2}023\right]}_{M}$ into twin state (red square).

**Figure 7:**
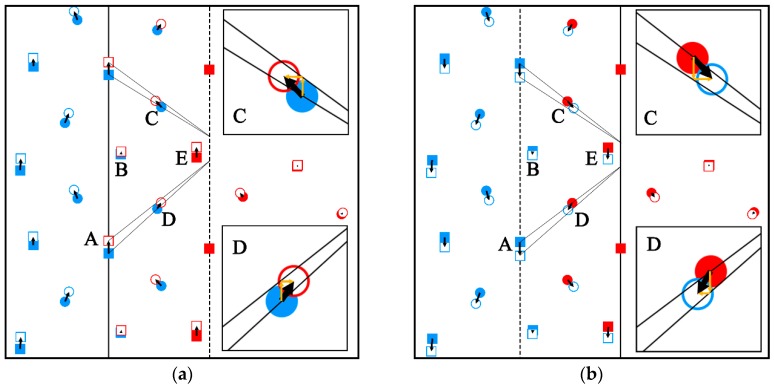
Illustration of atoms’ relative displacement during twin boundary migration under different shear directions. (**a**) [$\bar{1}011$] shear direction (**b**) [$10\bar{1}\bar{1}$] shear direction. The black dotted line implies the initial position of twin boundaries and the black solid lines imply the twin boundaries position after migration. The black arrows present the atom move directions. Orange arrows indicate the resolved directions. Squares and circles imply atoms on different {$\bar{1}2\bar{1}0$} planes. The full and hollow symbols indicate atoms before and after migration, respectively. Atom C, D are magnified in the black squares.

Furthermore, the relative displacement vectors of atoms in different TB structure during the TB migration process under different shear directions is presented in Figure 7. The solid and hollow symbols represent the atoms’ position before and after migration, respectively. Five atoms A, B, C, D, E were labeled to observe the atoms’ motion during the TB migration process, in which atoms A, B, E were in L_2_ layer beside the GTB structure and atoms C, D were in L_1_ layer beside the MSTB structure. When the external shear strain was along [$\bar{1}011$] direction (Figure 7a), atoms A, B, E moved along [$\bar{1}011$] direction, and the displacement value of atom A was approximately equal to the value of twin dislocation

(3) $$\left|b_{\mathrm{TD}}\right|=\left|\left(3-v^{2}\right)/\left(3+v^{2}\right)\;\left[\bar{1}011\right]\right|\approx 0.047\;\mathrm{nm}.$$

According to the moving characteristic on GTB, it could be concluded that

(4) $$b_{\mathrm{TD}}\to {\left[\bar{4}043\right]}_{M}+{\left[\bar{4}043\right]}_{T}.$$

Atom B, E moved to their proper position by the shuffling, their displacements were both smaller than b_TD_. As seen from the inset of Figure 7a, the displacement vectors of atoms C and D were smaller than b_TD_ and could be resolved into two components. The component paralleled to the TB was both along [$\bar{1}011$] direction, whose value was approximate to its theoretical shear value. The component perpendicular to the TB of atoms C and D moved along opposite direction by shuffling movement to accommodate the microstructural transformation of the MSTB during the migration process. In Figure 7b, when shearing along [$10\bar{1}\bar{1}$], the movement was totally opposite to that shearing along [$\bar{1}011$], but the move mode of atoms A–E was in the same process.

In the simulations, the magnitude of the shuffling was all smaller than b_T__D_, which was quite different to the shuffling-dominated mechanism [27] proposed by Li et al. They thought the shuffling was much greater than the twin dislocation and the twin dislocation was too small to impact on TB migration. However, they only considered the direct transformation from the parent to the twin without thinking about the TB’s contribution. Actually, the {$10\bar{1}2$} TB was not a single layer of {$10\bar{1}2$}, it contained several {$10\bar{1}2$} layers. As shown in Figure 6, the atoms’ movement could not adjust immediately in a single step, the {$10\bar{1}2$} TB transformed gradually. Thus, the closer the {$10\bar{1}2$} plane to TB, the more similar the configuration to TB’s, the smaller the shuffling magnitude would be. But in the process of TB migration, the shearing magnitude was constant, when TB migrated two {$10\bar{1}2$} layers, the shear displacement was equal to the b_TD_. That was to say, the transformation from parent to twin mentioned in shuffling-dominated mechanism was not the result of one slip of the TD, but twice, thrice, or even more times. It meant that the TB migration could not ignore the TD, on the contrary, the effect of shear caused by TD accounted for an important part. From the analysis of Figure 7 and the shear strain of the migration region, it was concluded that the TB migration was relative to both shear and atom shuffling. During TB migration, every atom was affected by the shear and different shear directions caused opposite move directions, leading to twinning or detwinning. The shuffling was only used for adjusting the GTB and MSTB structure evolution.

## 4. Conclusions

In this paper, an Mg crystal with two coherent twin boundaries under different shear directions and strain rates was studied by molecular dynamics simulation. The results showed that the twin structure would be thickened under [$\bar{1}011$] shear direction and shrunk with shearing in the opposite direction. The CRSS of {$10\bar{1}2$} TB migration increased with strain rate increasing. The ratios of TB migration distance and loading displacement ranged from 0.123 to 0.125 at all the strain rates, they were in excellent agreement with the twin shear value theory. According to the TB structure and atom displacement, twin dislocation was resolved by b_TD_ → ${\left[\bar{4}043\right]}_{M}$ + ${\left[\bar{4}043\right]}_{T}$. TB migration was achieved by both the shear and the atomic shuffling. Every atom was affected by the shear, different shear directions caused opposite atom move directions, leading to twinning or detwinning. The atom shuffling was only used for adjusting the GTB and MSTB structure evolution. Frankly speaking, the twinning boundary migration mechanism was discussed based on the coherent twin boundary. Whether it is suited for the incoherent TB would be a further research target in the subsequent works.

## Figures and Tables

**Table 1:** The ratio of loading displacement and migration distance under different shear directions.

Strain Rate (s^−1^)	Ratio of Loading Displacement and Migration Distance	
	[$\bar{1}011$]	[$10\bar{1}\bar{1}$]
10^7^	0.123	0.124
5 × 10^7^	0.124	0.124
10^8^	0.124	0.125
